# Isolation and characterization of *Streptococcus agalactiae* inducing mass mortalities in cultured Nile tilapia (*Oreochromis niloticus*) with trials for disease control using zinc oxide nanoparticles and ethanolic leaf extracts of some medicinal plants

**DOI:** 10.1186/s12917-024-04298-z

**Published:** 2024-10-15

**Authors:** Ebtsam Sayed Hassan Abdallah, Walaa Gomaa Mohamed Metwally, Soad Abdel Latief Hassan Bayoumi, Moataz Ahmed Mohamed Abdel Rahman, Mahmoud Mostafa Mahmoud

**Affiliations:** 1 Department of Aquatic Animal Medicine and Management, Faculty of Veterinary Medicine, Assiut University, Assiut, 71529 Egypt; 2 Poultry and Fish Diseases Department, Faculty of Veterinary Medicine, Minia University, Minia, 61519 Egypt; 3 Pharmacognosy Department, Faculty of Pharmacy, Assiut University, Assiut, 71526 Egypt; 4 Department of Behavior and Management of Animal Wealth, Faculty of Veterinary Medicine, Minia University, Minia, 61519 Egypt

**Keywords:** *Streptococcus agalactiae*, Nile tilapia, Phylogenetic analysis, RAPD analysis, Pathogenicity, Biofilm formation, Zinc oxide nanoparticles, Medicinal plants’ antibacterial activity

## Abstract

**Background:**

*Streptococcus agalactiae* (Group B streptococcus, GBS) induces a serious infection that can harm not only aquatic life but also humans and other animals. In a fish farm in southern Egypt, Nile tilapia (*Oreochromis niloticus*) has developed an epidemic with clinical symptoms resembling piscine streptococcosis.

**Results:**

Initial microscopic inspection of the affected fish brain and kidney indicated the presence of Gram-positive cocci. *S. agalactiae* was effectively isolated and identified using nucleotide homology of the *16S rRNA* and species-specific PCR. The partial *16S rRNA* sequence was deposited in the GenBank database at the NCBI and given the accession number MW599202. Genotyping using RAPD analysis indicated that the isolates in the present study belonged to the same genotypes and had the same origin. The challenge test, via immersion (9.2 × 10^7^, 9.2 × 10^6^, and 9.2 × 10^5^ CFU/ml for 1 h) or intraperitoneal injection (4.6 × 10^7^, 4.6 × 10^6^, and 4.6 × 10^5^ CFU/fish), elicited clinical symptoms resembling those of naturally infected fish with a mortality rate as high as 80%. The ability to create a biofilm as one of the pathogen virulence factors was verified. Zinc oxide nanoparticles and the ethanolic leaf extracts of nine medicinal plants demonstrated considerable antibacterial activities against the tested *S. agalactiae* strain with low minimum bactericidal concentrations (MBC) and minimum inhibitory concentrations (MIC). The ethanolic leaf extracts from *Lantana camara* and *Aberia caffra* showed potent antibacterial activity with MBC values of 0.24 and 0.485 mg/ml, and MIC values of 0.12 & 0.24 mg/ml, respectively.

**Conclusion:**

This study isolated *S. agalactiae* from *O. niloticus* mortalities in a fish farm in Assiut, Egypt. The pathogen persists in fish environments and can escape through biofilm formation, suggesting it cannot be easily eliminated. However, promising findings were obtained with in vitro control employing zinc oxide nanoparticles and medicinal plant extracts. Nevertheless further in vivo research is needed.

## Background

Streptococcosis is considered one of the most devastating bacterial diseases, causing economic losses in many fish species, especially those raised in warm water [1]. There have been numerous instances of streptococcosis in both cultured and wild marine fishes [2] as well as freshwater fish species worldwide [3, 4]. The disease’s most well-known clinical signs are “pop eye” and erratic swimming [5]. Although many bacterial pathogens have been linked to fish streptococcosis, the Gram-positive bacteria *Streptococcus agalactiae*, *S. iniae*, *S. parauberis*, and *S. dysgalactiae* are by far the most prevalent causative agents of these diseases all over the world [3, 5]. *Streptococcus agalactiae* (Lancefield group B streptococci, GBS) is an emerging zoonotic pathogen originally known as *S. difficile* [6], is linked to diseases not only in fish but also in humans, dogs, cows, horses, and other animals [7].

Nile tilapia (*Oreochromis niloticus*) is a prominent freshwater aquaculture species globally. Since intensive tilapia farming has supplanted traditional tilapia farming, it is more vulnerable to several infectious diseases, such as streptococcosis caused by *S. agalactiae*, which causes mass mortalities in *O. niloticus* aquaculture with huge economic losses worldwide [1, 8–17]. Traditional phenotypic methods in conjugation with molecular methods were used to identify this pathogen. In addition, DNA-based typing techniques such as random amplification of polymorphic DNA (RAPD), which is a simple technique that can provide a sufficient assay for polymorphism [18], have been used to genotype *S. agalactiae* isolates originating from different sources. The fecal-oral pathway is the primary means of streptococcosis transmission [19]. Diseased fish can harbor bacteria in their excrement that can live in water and spread to healthy fish [20]. Loss of appetite, unilateral or bilateral exophthalmos (pop-eye), corneal opacity, hemorrhage on the skin, base of the fins, or around the eyes, accumulation of serosanguineous fluid in the abdominal cavity, meningitis, and neurological signs like circling, swirling, or disorientation are the most common gross findings in tilapia infected with *S. agalactiae* [11, 12, 14, 15, 21]. It is commonly known that the pathogenesis and persistence of certain bacteria and bacterial diseases depend on biofilm production [22]. These ubiquitous microbiological communities, embedded in adherent extracellular matrices, have a significant and possibly contradictory function in aquaculture. Additionally, biofilms have the potential to serve as a reservoir for pathogenic microorganisms, protecting and harboring them to raise the risk of recurrent infections [23]. A disease outbreak with fish exhibiting septicemic symptoms has been recently recorded in a Nile tilapia farm in Assiut City, Egypt, resulting in substantial mortalities and financial losses. Therefore, the present study was directed to investigate and characterize the disease-causing agent. Trials to control the disease, employing ethanolic leaf extracts of some medicinal plants as well as zinc oxide nanoparticles, have also been conducted.

## Methods

### Fish

**Fig. 1:**
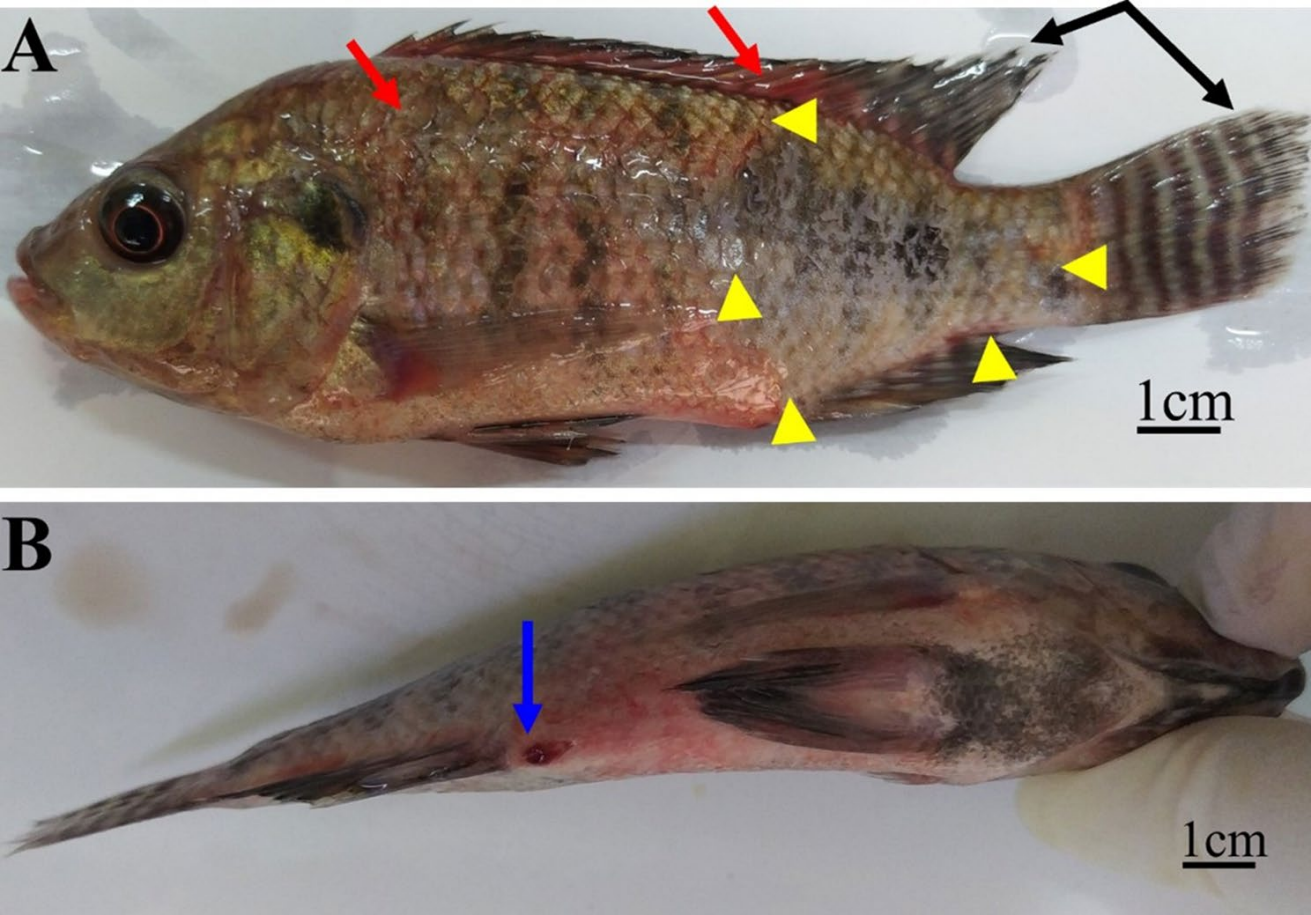
Moribund Nile tilapia (*Oreochromis niloticus*) naturally infected with *Streptococcus agalactiae* showing **A**: dark coloration with severe hemorrhages throughout the body (red arrow) with a large area of scale loss (red arrow-head), hemorrhages at the base of the fins, frayed fins (black arrow) and **B**: hemorrhagic vent (blue arrow)

Moribund Nile tilapia (*n* = 60), exhibiting evidence of septicemia (Fig. 1), were caught in the summer of 2020 from an aquaculture facility at the Faculty of Veterinary Medicine, Assiut University, Assiut, Egypt. This facility had experienced significant mortalities with a septicemic picture and some other symptoms on the dead fish, including dark coloration, hemorrhages distributed throughout the body and at the base of the fins, corneal opacity, unilateral or bilateral exophthalmia, abdominal distension, and skin ulceration with scale loss. Moribund fish were transported directly to the fish diseases laboratory at Assiut University. Their average standard length and average body weight were 13.5 ± 1.5 cm and 45 ± 10 g, respectively. The fish were humanely euthanized using clove oil [24] for tissue sampling, and handled in accordance with the standard protocol approved by Minia University, Faculty of Veterinary Medicine Ethics Committee for Animal Use and Care (Number IRB-FVM-MU-2020-54, date 4.3.2020).

### Isolation and identification of the causative agent

From the sampled fish, Gram-stained impression smears were made from the anterior kidney and brain. Tryptic soy agar (TSA; Biolife), brain heart infusion agar (BHIA; Himedia), and Streptococcus selective agar (SSA; Himedia) were used to isolate the causative agent. Plates were then incubated at 28 °C for 48 h. Purified dominant isolates were preserved at − 80 °C in BHI broth containing 25% glycerol for further characterization [25].

For the colony morphology study, bacteria were streaked on different culture media (BHIA, TSA, and SSA), incubated at 28 °C for 24 h, and 48 h, photographed, and measured using a Leica Microsystem (Switzerland; version 3.4.0). Subsequently, bacterial isolates were characterized phenotypically, physiologically, and biochemically using conventional methods (listed in Table 1). The tests included Gram-stain, motility in sulfide indole motility medium, hemolysis on 5% sheep blood agar, cytochrome oxidase, catalase, esculin hydrolysis on bile esculin slants, and H_2_S production in TSI slants. The survival of the isolates at different temperatures (10, 15, and 42 °C), as well as their tolerances to various salinities (1.5, 4.5, 5, 6, and 6.5%), have been determined. To investigate the period within which the bacteria can survive in sterile freshwater, the tested isolate was inoculated in 0.2 μm-filter sterilized fresh water and incubated at various temperatures (15, 28, and 35 °C). Thereafter, bacterial counts (triplicates/isolate) were performed daily until the complete disappearance of bacteria for three consecutive days.

**Table 1:** Phenotypic characteristics of *Streptococcus agalactiae* (MW599202) strains isolated from Nile tilapia (*Oreochromis niloticus*) mass mortalities using conventional tests

Characteristics	*Streptococcus agalactiae*					
	MW599202	ESHA Strept.65	ESHA Strept.66	ESHA Strept.68	AH2 strain AH Al-Harbi [8]	K Wang, D Chen, L Huang, H Lian, J Wang, D Xiao, Y Geng, Z-x Yang and W-m Lai [15] 8 isolates
Gram stain	+	+	+	+	+	+
Catalase	-	-	-	-	-	-
Oxidase	-	-	-	-	-	NA
Motility (Sulfide indole motility semisolid medium)	-	-	-	-	-	NA
Hemolysis on 5% sheep blood agar	β	β	β	β	-	-
H_2_S production (Triple sugar iron agar; TSI)	-	-	-	-	-	NA
Growth on TSI	A/A	A/A	A/A	A/A	A/A	NA
Growth in bile esculin agar	-	-	-	-	-	-
Growth at						
10 °C	-	-	-	-	-	-
15 °C	+	+	+	+	+	NA
42 °C	+	+	+	+	+	NA
NaCl tolerance						
1.5%	+	+	+	+	NA	NA
4%	+	+	+	+	NA	NA
5%	+	+	+	+	+	NA
6%	+	+	+	+	NA	NA
6.5%	-	-	-	-	-	-
Acid from carbohydrate						
Ribose	+	+	+	+	+	+
Lactose	-	-	-	-	-	+
Xylose	-	-	-	-	-	NA
Arabinose	+	+	+	+	NA	-
Maltose	+	+	+	+	+	NA
Mannitol	-	-	-	-	-	-
Inulin	-	-	-	-	NA	-
Raffinose	-	-	-	-	-	-
Trehalose	+	+	+	+	+	+
Sorbitol	-	-	-	-	-	-
Sucrose	+	+	+	+	+	NA
Salicin	-	-	-	-	-	NA
Na pyruvate	+	+	+	+	NA	NA

+: Positive reaction, -: Negative reaction, NA: no data available

To characterize the isolates molecularly, colonies (grown on BHIA) were picked and the whole genomes were extracted using the CTAB method, as previously described (Abdallah et al., 2018). Then, a nanodrop spectrophotometer (Implen GmbH, Germany) was used to measure DNA concentration and purity at an optical density (OD) of 260 nm and a relative OD of 260/280 nm, respectively. Until used, DNA samples were stored at − 20 °C. The universal primers 27F and 1492R [26] were employed in a polymerase chain reaction (PCR) to amplify the hypervariable 1500 bp segment of the *16S rRNA*. A total of 50 µl volume was used for the PCR reactions, which consisted of 25 µl MyTaq red mix (Bioline, UK), 2 µl of each primer, 4 µl template DNA (containing 100 ng of the whole bacterial genome), and 17 µl H_2_O (RNase /DNase free). The Veriti thermal cycler (Applied Biosystems, USA) was used to perform PCR amplification with an initial denaturation step at 95 °C for 5 min, followed by 35 cycles of denaturation at 94 °C for 1 min, annealing at 55 °C for 1 min, extension at 72 °C for 1.5 min, and a final extension step at 72 °C for 10 min. Additionally, a *Streptococcus agalactiae*-specific PCR was carried out following the method of G Martinez, J Harel and M Gottschalk [27], using a species-specific primer set, (F1: 5`-GAGTTTGATCATGGCTCAG-3` and 1MOD: 5`-ACCAACATGTGTTAATTACTC-3`) targeting the *16S rRNA* gene (220 bp). The PCR products were electrophoresed on a 1.5% agarose gel, stained with ethidium bromide, and visualized using a UV transilluminator (MultiDoc- It, UVP, UK). The size of the PCR products was determined using a 100-bp DNA ladder. Subsequently, the PCR products from the gel were purified using the Zymoclean Gel DNA Recovery Kit (Zymo Research, USA) before being sequenced with the same amplification primers. Similarities to other related published sequences were assessed using the Basic Local Alignment Search Tool (BLAST) (http://www.ncbi.nlm.nih.gov/BLAST/*).*

### Phylogenetic analysis

Trimming the *16S rRNA* gene sequences obtained in this investigation was done using DNA Baser (version 5.15.0). The resulting *16S rRNA* gene sequences were compared to closely comparable sequences in the GenBank and then uploaded to the GenBank to get accession numbers. *Lactococcus garvieae* (MF351803.1) served as the outgroup in this study. The Maximum Likelihood approach and the Tamura-Nei model [28], which used the random stepwise addition of 1000 replicates, were used to infer the evolutionary history. The tree that has the highest log likelihood (-4024.82) is displayed. By automatically applying Neighbor-Join and BioNJ algorithms to a matrix of pairwise distances calculated using the Tamura-Nei model and then choosing the topology with the highest log likelihood value, the initial tree(s) for the heuristic search were created. The tree was depicted to scale (next to the branches), with branch lengths measured in the number of substitutions per site. The proportion of sites where at least 1 unambiguous base is present in at least 1 sequence for each descendent clade was shown next to each internal node in the tree. There were 17 nucleotide sequences in this investigation. Codon positions included were 1st + 2nd + 3rd + Noncoding. Gaps and missing data were removed from all positions (complete deletion option). The final dataset contained 1513 positions altogether. MEGA11 version 11.0.13 was used to conduct an evolutionary analysis [29]. A TIF file was created when the tree was modified in Microsoft PowerPoint 365. Additionally, the Maximum Composite Likelihood model [30] was used to calculate the intraspecific and interspecific divergence distances among 16 *Streptococcacea 16S rRNA* gene sequences, including the isolate used in the current investigation.

### Fingerprinting and genetic relatedness among isolates

According to the manufacturer’s recommendation, a Ready-To‐Go Random Amplified Polymorphic DNA (RAPD) PCR Analysis Kit (GE Healthcare, UK) with six primers (P1 to P6) was used to perform the RAPD PCR analysis for representative isolates (*n* = 4), as previously described [18]. Briefly, the PCR mixture of 25 µL contained Ready-To-Go RAPD analysis beads, 25 pmol of a single RAPD primer, 50 ng of template DNA, and nucleases-free distilled water. The bead contained thermo-stable polymerase, dNTPs (0.4 mM each dNTP in a 25 µl reaction volume), BSA (2.5 µg) and buffer (3 mM MgCl_2_, 30 mM KCl and 10 mM Tris, pH 8.3 in a 25 µl). Six primers (P1-P6; GE Healthcare, UK) were used in this study. Each primer is a 10-mer of arbitrary sequence: P1 (5′-GGTGCGGGAA-3′), P2 (5′-GTTTCGCTCC-3′), P3 (5′-GTAGACCCGT-3′), P4 (5′-AAGAGCCCGT-3′), P5 (5′-AACGCGCAAC-3′) and P6 (5′-CCCGTCAGCA-3′). PCR was performed using a Veriti 96-well thermal cycler (Applied Biosystems, USA). PCR conditions included 1 cycle of 95 °C for 5 min, followed by 45 cycles of 95 °C for 1 min, 36 °C for 1 min and 72 °C for 2 min. The PCR products were electrophoresed using 2% agarose gel in Tris-acetate EDTA (TAE) buffer, stained with 0.05 µg/ml ethidium bromide (Serva, Germany), and visualized using UV transillumination (MultiDoc- It, UVP, UK). The size of the PCR products was determined using a 100 bp DNA ladder H3 RTU (GeneDireX).

### Challenge test

One hundred and twenty apparently healthy *O. niloticus*, with an average body weight of 25 ± 3 g and an average standard length of 9.0 ± 0.3 cm, were utilized. Before experimental infection, the fish were kept in a flow-through system (200-liter glass aquaria), fed commercial feed, and given two weeks of acclimation following the method of Ellsaesser & Clem [31]. Feeding was halted two days before the experimental infection. The dissolved oxygen content was kept between 6.0 and 7.0 mg/L, the pH was between 7.2 and 7.5, and the water’s temperature was 28 ± 1 °C. For verification, five fish were randomly selected and underwent comprehensive clinical, bacteriological, and molecular analysis to confirm their freedom from *S. agalactiae*, as outlined by E Soto, M Zayas, J Tobar, O Illanes, S Yount, S Francis and MM Dennis [32]. The randomization of fish into groups followed the guidelines set forth by The Animals in Research Reporting In Vivo Experiments 2 (ARRIVE 2) guidelines [33]. *S*. *agalactiae* (ESHA-*Strept.* 1), isolated in the present study, was used. The fish were divided into 8 groups of 15 fish each. The first three groups received intraperitoneal (IP) injections [18, 34] of 0.05 ml bacterial suspension in PBS (4.6 × 10^7^, 4.6 × 10^6^, and 4.6 × 10^5^ CFU/fish, respectively). Fish in the fourth, fifth, and sixth groups, were immersed in water containing 9.2 × 10^7^, 9.2 × 10^6^, and 9.2 × 10^5^ CFU/ml for one hour before being transferred to corresponding 200 L aquaria containing chlorine-free water. The seventh (sham control) group was IP injected with 0.05 ml of sterile PBS. As an absolute control, the eighth group, was kept unexposed to any experimental interference to ensure that the mortalities were only due to the pathogen and not to other environmental factors. The experimental infection was conducted in triplicates under static conditions. Fish were monitored daily for 15 days, and any clinical signs and mortalities were recorded. Recently dead fish were subject to bacterial re-isolation. Simple linear regression analysis in GraphPad Prism 8 (version 8.4.3 (686) June 2020) was used to analyze the survival rates after 15 days. Then, the survivors were humanely euthanized using clove oil [24] for tissue sampling. The pathogen was then retrieved from the kidney, and brain on BHIA.

### Biofilm detection and quantification

To determine the ability of biofilm generation, representative isolates (*n* = 7) were tested as described by ESH Abdallah, MM Mahmoud and IR Abdel-Rahim [35] with some modifications. Briefly, the bacterial count was adjusted to around 7 × 10^5^ CFU/ml in BHI broth (BHIB). Two hundred microliters of the bacterial suspension in BHIB per well were added to the 96-well polyvinyl chloride (PVC) microtiter plate, with twelve replicates. The negative control consisted of wells containing uninoculated media. The plates were then incubated at 28 °C for 48 h. Biofilm quantification was performed by the crystal violet assay, according to T-J Kim, BM Young and GM Young [36]. Biofilm development was quantified using monitoring the OD_630_ values with an ELx808™ microplate reader running Gene 5 software (Bio‐Tek, USA). Following the procedures outlined by S Stepanović, D Vuković, I Dakić, B Savić and M Švabić-Vlahović [37], the tested strains were categorized into four groups according to their OD values, for biofilm interpretation. This was carried out after subtracting the control OD, resulting in classifications of: no biofilm producer; weak biofilm producer; moderate biofilm producer, and strong biofilm producer.

### Antimicrobial activity of medicinal plants

The assay was performed using sterile polystyrene 96-well microtiter plates as previously described by JR Soberón, MA Sgariglia, DA Sampietro, EN Quiroga and MA Vattuone [38], with minor modifications. Briefly, the leaves of *Aberia caffra* Hook. f. & Harv., *Azadirachta indica* L., *Dodonaea viscosa* L., *Ficus nitida* L., *Lanatana camara* L., *Myrtus communis* L., *Olea europaea* L., *Ruta graveolens*, and *Schinus terebinthifolius* Raddi during the flowering stage were extracted using 70% ethanol [39] and then dissolved in dimethylsulfoxide (DMSO) to give a concentration of 200 mg/ml. In the first well, 100 µl of each plant extract was added to 100 µl of sterile double-strength BHIB. A two-fold serial dilution of each extract was done, resulting in a concentration range of 250–0.122 mg/ml. Thereafter, each well received 100 µl of a bacterial suspension in BHIB containing 1 × 10^5^ CFU/ml. To exclude any potential antibacterial effects of the solvent, bacterial growth controls were made by adding DMSO to the first well. The highest concentration of DMSO (25%) was in the first well and decreased two-fold in each subsequent well, and the bacterial growth was never inhibited by 25% DMSO. Sterility controls were created by using just sterile, uninoculated BHIB. Each assay was carried out in triplicate. For 24 h, the plates were then incubated at 28ºC. The plant extract’s minimum inhibitory concentration (MIC) was estimated as that which inhibited bacterial growth. Twenty microliters from each well were aseptically aspirated, inoculated onto BHIA, and incubated at 28ºC for 48 h to determine the minimum bactericidal concentration (MBC). Aliquots obtained from the growth control wells were used as bacterial viability controls. The MBC was designated as the lowest concentration of plant extract that showed no bacterial growth.

### Antibacterial activity of zinc oxide nanoparticles (ZnO NPs)

Zinc oxide nanoparticles (ZnO NPs, Sigma Aldrich, < 35 nm average particle size) were tested for their antibacterial activities in sterile polystyrene 96-well plates using the approach of ESH Abdallah, MM Mahmoud and IR Abdel-Rahim [35], with some modifications to allow for the growth of *S. agalactiae*. Cells of *S. agalactiae* (MW599202) were cultured in BHIB at 28 °C for 24 h, and the cell number was adjusted to 1.5 × 10^6^ CFU/ml using a ten-fold serial dilution [40]. In the first well of a 96-well microtiter plate, 100 µl of sterile double-strength BHIB were introduced together with 100 µl containing 170 mg of ZnO NPs. A two-fold serial dilution of ZnO NPs was done. *S. agalactiae* (100 µl) was added to each concentration of ZnO NPs in BHIB at a ratio of 1:1 (v/v), resulting in final concentrations of 42.5, 21.25, 10.625, 5.312, 2.65, 1.32, 0.66, 0.33, 0.17, 0.08, 0.04, and 0.02 mg /well, and incubated at 28 °C for up to 72 h. The negative control (0 mg/well) contained just *S. agalactiae*- inoculated BHIB. Following the procedure previously described by AA Miles, SS Misra and JO Irwin [40], the total *S. agalactiae* viable cell count was estimated at 2, 4, 18, 24, 48, and 72 h post-inoculation. To count the developed colonies, 20 µl from each treatment was aseptically aspirated, serially diluted ten times in sterile PBS, dropped onto BHIA plates, and incubated at 28 °C for 48 h. MBC was determined to be the ZnO NPs concentration at which *S. agalactiae* cell growth was completely inhibited.

### Statistical analysis

The one-way analysis of variance (ANOVA; Kruskal-Wallis test) was used to analyze the quantification of biofilms. The data on the antibacterial action of ZnO NPs on the tested strain were analyzed using a two-way ANOVA. To assess the data on survival rates in the challenged fish, simple linear regression was applied. Prism^®^ 8 software (version 8.4.3) programmed onto Graph Pads was used for all analyses. Each result is the mean of three replicates ± the standard error of the mean (SEM) value. A probability of 5% or less was considered a significant difference.

## Results

### Isolation and identification of the causative agent

Naturally infected Nile tilapia had a dark overall color, significant hemorrhages throughout the body, on the head, and at the base of the fins, along with areas of ulceration and scale loss, frayed fin tips, and hemorrhagic protruded vents (Fig. 1). Some fish also exhibited exophthalmia and intraocular hemorrhages. Internally, they had splenomegaly, hemorrhagic friable livers, and hemorrhagic brains. When the moribund fish’s brain and anterior kidney impression smears were examined under a microscope, numerous Gram-positive cocci were found.

**Fig. 2:**
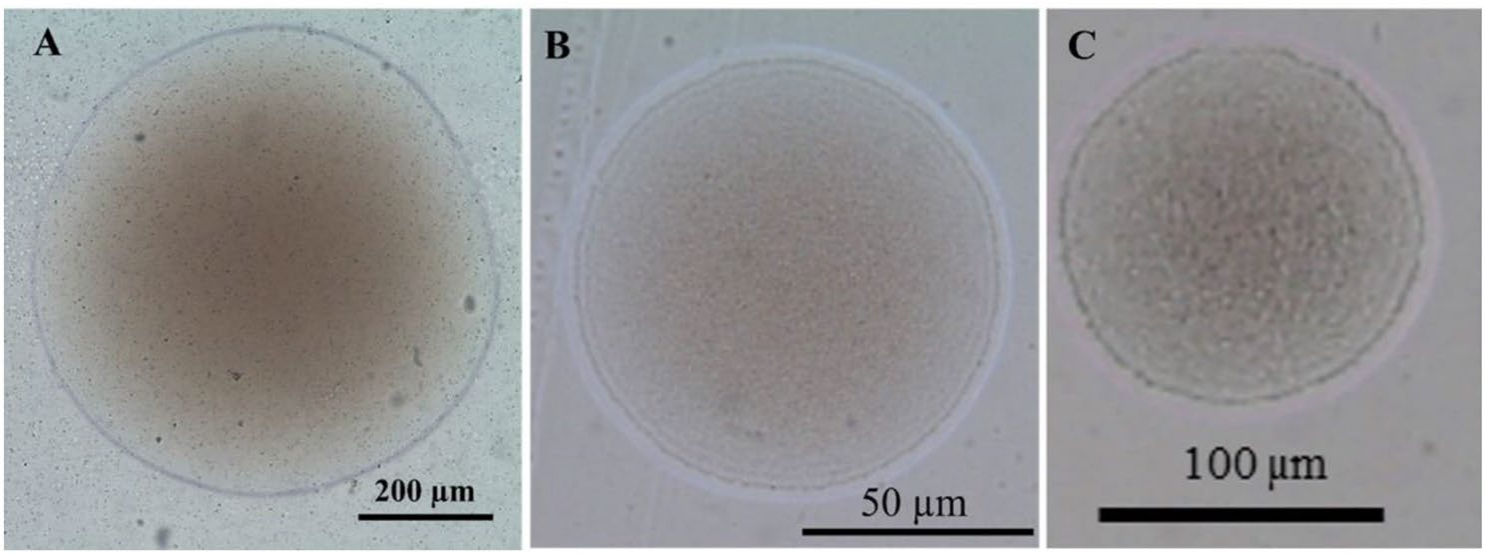
Colony morphology of *Streptococcus agalactiae* isolated from Nile tilapia (*Oreochromis niloticus*) on three different media: brain heart infusion agar (**A**), trypticase soy agar (**B**), and streptococcal selective agar (**C**). Plates were incubated at 28 °C for 24 hours

The isolated bacterial colonies emerged as tiny white opaque polymorphic smooth-edged spherical colonies with an elevated center on BHIA, TSA, and SSA plates (Fig. 2). These colonies’ density increased over time, with an average diameter of 0.7 ± 0.02 mm and 0.9 ± 0.2 mm after an incubation period of 24 and 48 h, respectively, at 28 °C on BHIA (Fig. 2A). However, after being incubated at 28 °C for 24 and 48 h, respectively, the colony seemed smaller and slower-growing on TSA, with an average colony diameter of 0.2 ± 0.08 mm and 0.7 ± 0.1 mm. Following 24 and 48 h of incubation at 28 °C, smaller and lighter-colored colonies with an average diameter of 0.1 ± 0.02 mm and 0.2 ± 0.04 mm, respectively, formed on SSA (Fig. 2C). The phenotypic and biochemical characteristics are listed in Table 1.

**Fig. 3:**
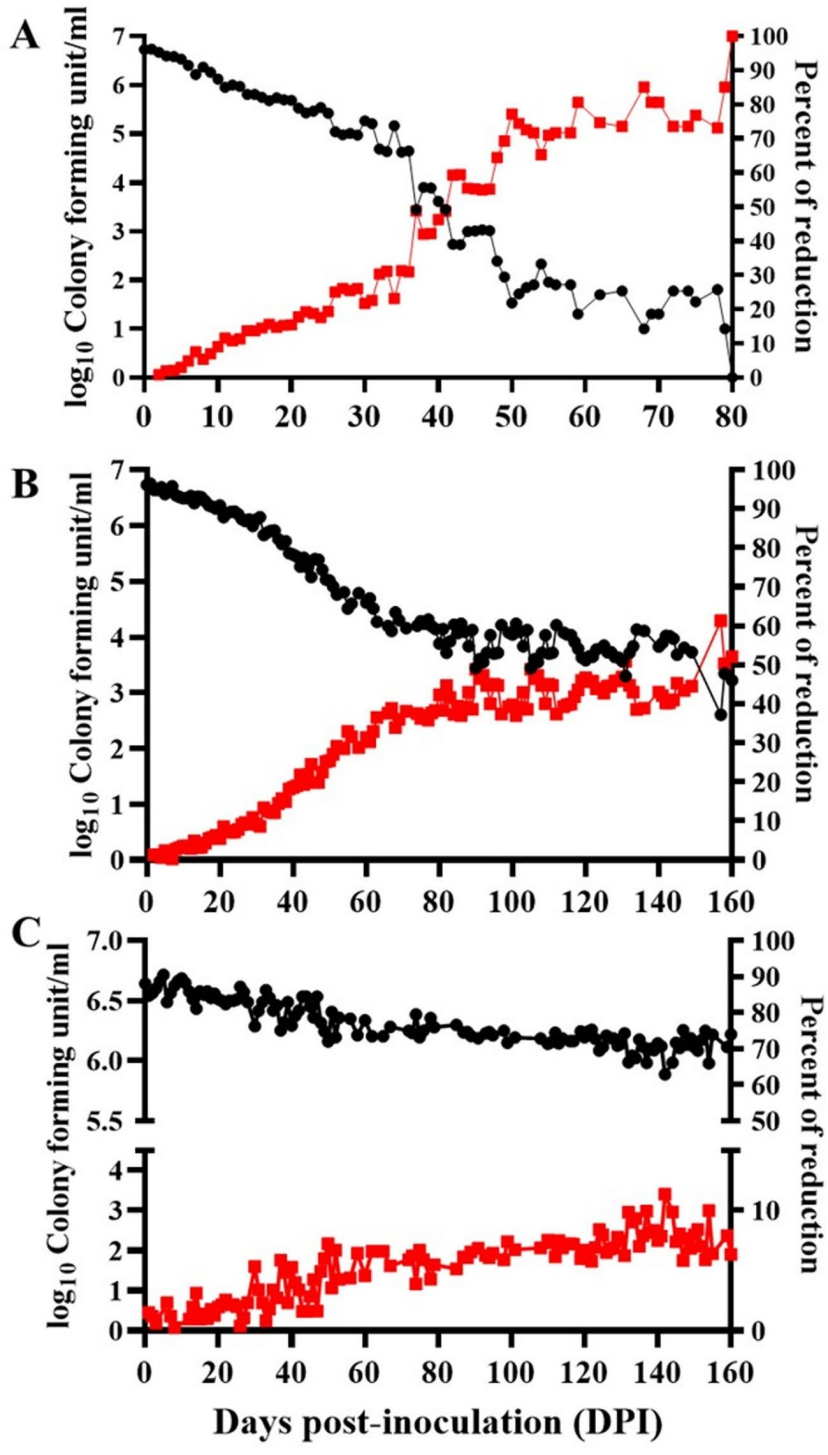
Survivability of *Streptococcus agalactiae* (MW599202) in sterile fresh water at different incubating temperatures, where (**A**) incubated at 35 °C, (**B**) incubated at 28 °C) incubated at 15 °C. Viable bacteria were detected using trypticase soy agar

In sterile fresh water, *S. agalactiae* (MW599202) may survive for long periods (80, 160, 160) days post-inoculation (DPI) and be incubated at 35 °C, 28 °C, and 15 °C, respectively (Fig. 3). For the incubating temperature of 35 °C, the viable bacterial count decreased: the first log_10_ (5.7) was after 16 DPI, with a 14.6% reduction; the second log_10_ (4.9) reduction was after 27 DPI, with a 26% reduction; the third log_10_ (3.6) reduction was at 40 DPI, with a 46.3% reduction; the fourth log_10_ (3.0) reduction was at 47 DPI, with a 55.2% reduction; the fifth log_10_ (1.9) reduction was at 58 DPI, with a 71.7% reduction; and the sixth log_10_ (1.0) reduction was at 79 DPI, with a 85.1% reduction. The bacteria completely vanished from the inoculated sterile fresh water at 80 DPI and incubated in a static condition at 35 °C with a 100% reduction in the viable bacterial count (Fig. 3A). The size of the recovered bacterial colony was much smaller than that of the bacteria cultivated at 28 °C or 15 °C. The first log_10_ reduction (5.7) was at 36 DPI with a percent reduction of 14.5 for the bacteria incubated at 28 °C; the second log_10_ reduction (4.7) was at 52 DPI with a percent reduction of 29.2; and the third log_10_ reduction (3.7) was at 149 DPI with a percent reduction of 44.6; that continued to be 3.3 log_10_ reduction at 160 DPI with a 52.1% reduction (Fig. 3B). However, the first log_10_ drop in the total viable bacterial counts has not been detected up to 160 DPI (6.2 log_10_), and incubation in a static setting at 15 °C has only a reduction percent of 6.4 (Fig. 3C).

### Phylogenetic analysis

**Fig. 4:**
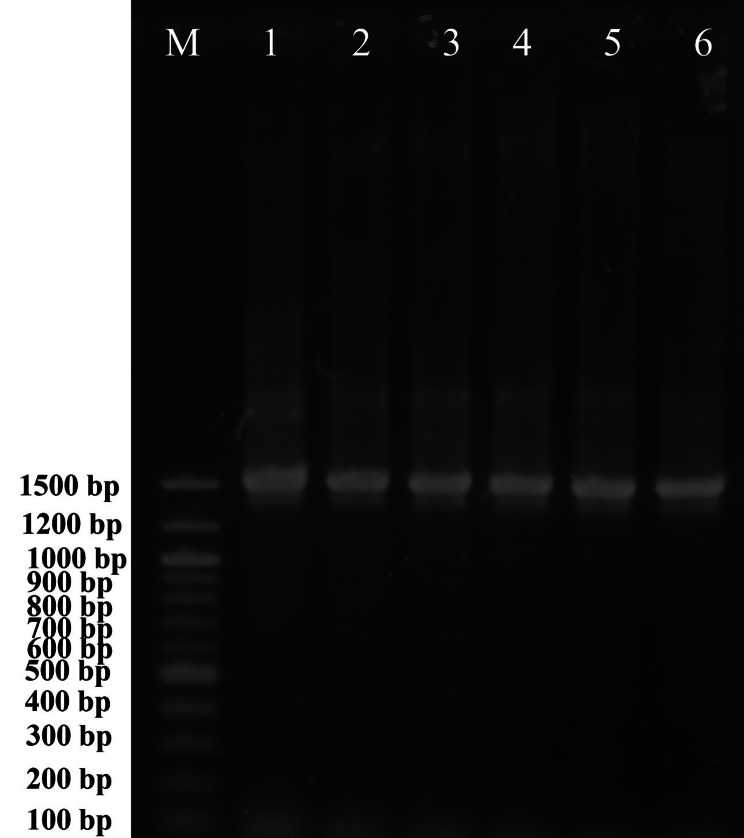
Molecular identification of *Streptococcus agalactiae* isolates from naturally infected Nile tilapia (*Oreochromis niloticus*) based on the amplification of the *16S rRNA* gene using agarose gel electrophoresis (1.5%). M: 100-bp DNA ladder. 1–6 tested samples

Following the sequencing of the *16S rRNA* region (Fig. 4), BLAST analysis using the sequence from the current investigation revealed 100% identity with 100% coverage with the *S. agalactiae* isolate HQ658087 isolated from Ya-fish (*Schizothorax prenanti*) in China; It also showed 100% identity with 99% coverage with *S. agalactiae* isolates MZ955884, a human pathogen in Poland, NR 040821, the type strain (ATCC 13813 strain JCM 5671) isolated from milk in the USA, KM209200 isolated from Tilapia in Indonesia, KM209201 isolated from Tilapia in Indonesia, OP290419 isolated from Tilapia in Vietnam, and OL636133, a medical isolate. The present study partial *16S rRNA* sequence was deposited in the GenBank on the NCBI under accession number MW599202.

**Fig. 5:**
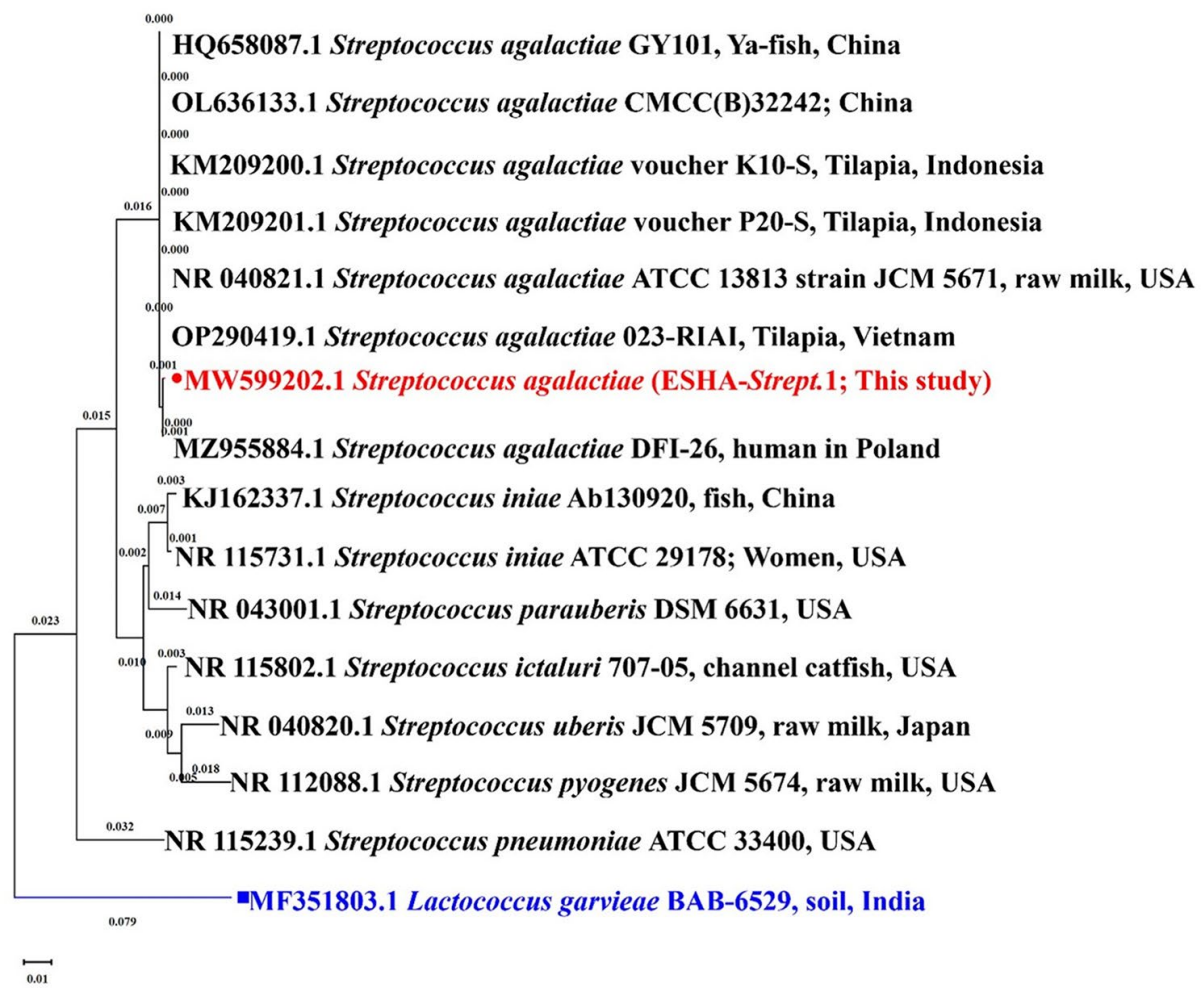
The phylogenetic tree depicts the relationship between *Streptococcus agalactiae* (MW599202 in red) isolated from naturally infected Nile tilapia (*Oreochromis niloticus*) and other *Streptococcus sp*. strains based on *16S rRNA* gene sequences. The tree was rooted to *Lactococcus garvieae* MF108375 as the outgroup (in blue). The tree was created by utilizing the maximum likelihood model. The bootstrap values (given as a percentage of 1000 replicates) are displayed at each branch nod (bar = 0.01 substitutions per nucleotide)

**Fig. 6:**
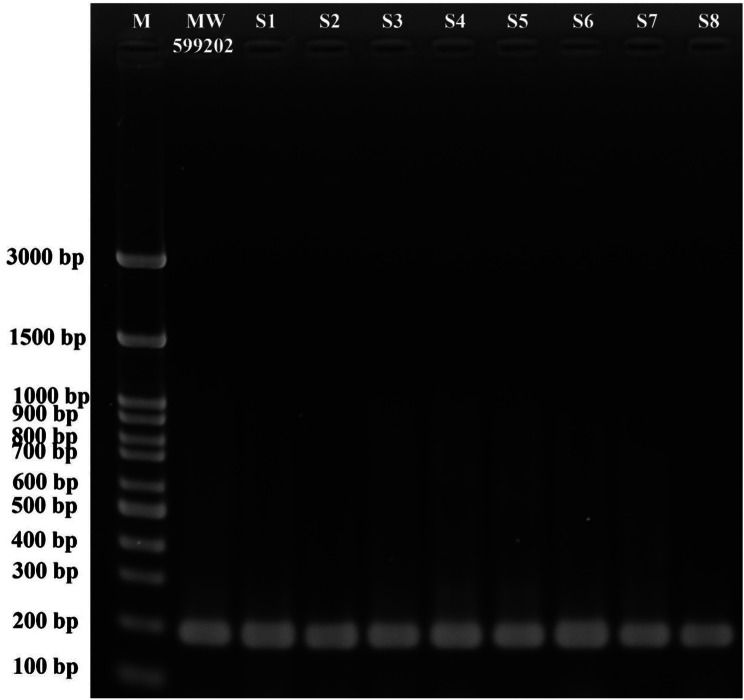
Molecular identification of *Streptococcus agalactiae* from naturally infected Nile tilapia (*Oreochromis niloticus*) to the species level, based on the amplification of the 220 bp region of *16S rRNA* gene using agarose gel electrophoresis (1.5%). M: 100 bp DNA ladder. Lane 2 *S. agalactiae* sequenced in this investigation (MW599202), and S1-S8 are tested isolates

Maximum likelihood analysis, using the Mega-X program, showed a close relationship between this research isolate (MW599202) and other *S. agalactiae* strains, suggesting they descended from a monophyletic clade (Fig. 5). Additionally, all the current investigation isolates were confirmed to be *S. agalactiae* using the *S. agalactiae-*specific primer that amplifies 220 bp of the *16S-rRNA* region (Fig. 6). Furthermore, the *16S-rRNA p*-distances between *S. agalactiae* strains (*n* = 8; including the current isolate) and other *Streptococcaceae* included in this investigation were significantly higher (0.020–0.141; Table 2).

**Table 2:** Intraspecific and interspecific divergence distance among *16 S rRNA* gene sequences of *Streptococcacea* and the current study isolate; *Streptococcus agalactiae* MW599202 that was isolated from mass mortalities of cultured Nile tilapia (*Oreochromis niloticus*)

	MW599202.1 *S. agalactiae*	HQ658087.1 *S. agalactiae*	MZ955884.1 *S. agalactiae*	OL636133.1 *S. agalactiae*	OP290419.1 *S. agalactiae*	KM209200.1 *S. agalactiae*	KM209201.1 *S. agalactiae*	NR_040821.1 *S. agalactiae*	KJ162337.1 *S. iniae*	NR_115731.1 *S. iniae*	NR_043001.1 *S. parauberis*	NR_043001.1 *S. parauberis*	NR_040820.1 *S. uberis*	NR_112088.1 *S. pyogenes*	NR_115802.1 *S. ictaluri*	MF351803.1 *L. garvieae*
MW599202.1 *S. agalactiae*																
HQ658087.1 *S. agalactiae*	0.000															
MZ955884.1 *S. agalactiae*	0.000	0.001														
OL636133.1 *S. agalactiae*	0.000	0.000	0.001													
OP290419.1 *S. agalactiae*	0.000	0.000	0.001	0.000												
KM209200.1 *S. agalactiae*	0.002	0.000	0.001	0.000	0.000											
KM209201.1 *S. agalactiae*	0.002	0.000	0.001	0.000	0.000	0.000										
NR_040821.1 *S. agalactiae*	0.002	0.000	0.001	0.000	0.000	0.000	0.000									
KJ162337.1 *S. iniae*	0.027	0.029	0.029	0.029	0.029	0.029	0.029	0.029								
NR_115731.1 *S. iniae*	0.020	0.028	0.028	0.028	0.028	0.033	0.033	0.033	0.004							
NR_043001.1 *S. parauberis*	0.022	0.035	0.035	0.035	0.035	0.040	0.040	0.040	0.020	0.021						
NR_115239.1 *S. pneumoniae*	0.033	0.055	0.056	0.055	0.055	0.059	0.059	0.058	0.053	0.055	0.060					
NR_040820.1 *S. uberis*	0.035	0.046	0.046	0.046	0.046	0.046	0.046	0.046	0.035	0.030	0.030	0.061				
NR_112088.1 *S. pyogenes*	0.035	0.032	0.032	0.031	0.031	0.037	0.037	0.036	0.038	0.041	0.038	0.062	0.031			
NR_115802.1 *S. ictalurid*	0.035	0.034	0.034	0.034	0.034	0.038	0.038	0.038	0.025	0.020	0.024	0.061	0.022	0.025		
MF351803.1 *L*. *garvieae*	0.141	0.104	0.104	0.104	0.104	0.134	0.134	0.133	0.100	0.130	0.128	0.138	0.128	0.130	0.135	

### Fingerprinting and genetic relatedness among representative isolates

**Fig. 7:**
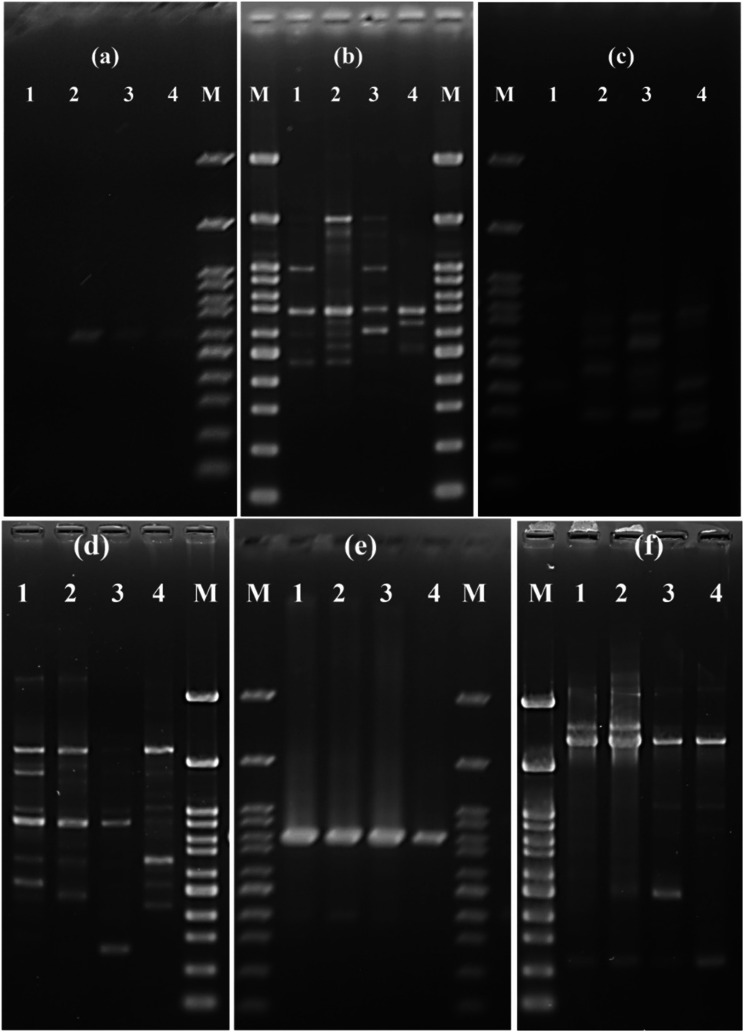
Random amplified polymorphic DNA (RAPD) PCR analysis of *Streptococcus agalactiae* isolates derived from naturally infected Nile tilapia (*Oreochromis niloticus*) mass mortalities. Primers 1, 2, 3, 4, 5, and 6 are denoted by the letters **a, b, c, d, e,** and **f**. M, Molecular marker (100 bp). 1–4 are the tested *S. agalactiae* isolates

The RAPD analysis of the six different primers (P1 to P6) showed that all of the tested isolates had essentially the same banding pattern with no evident polymorphism (Fig. 7).

### Challenge test

**Fig. 8:**
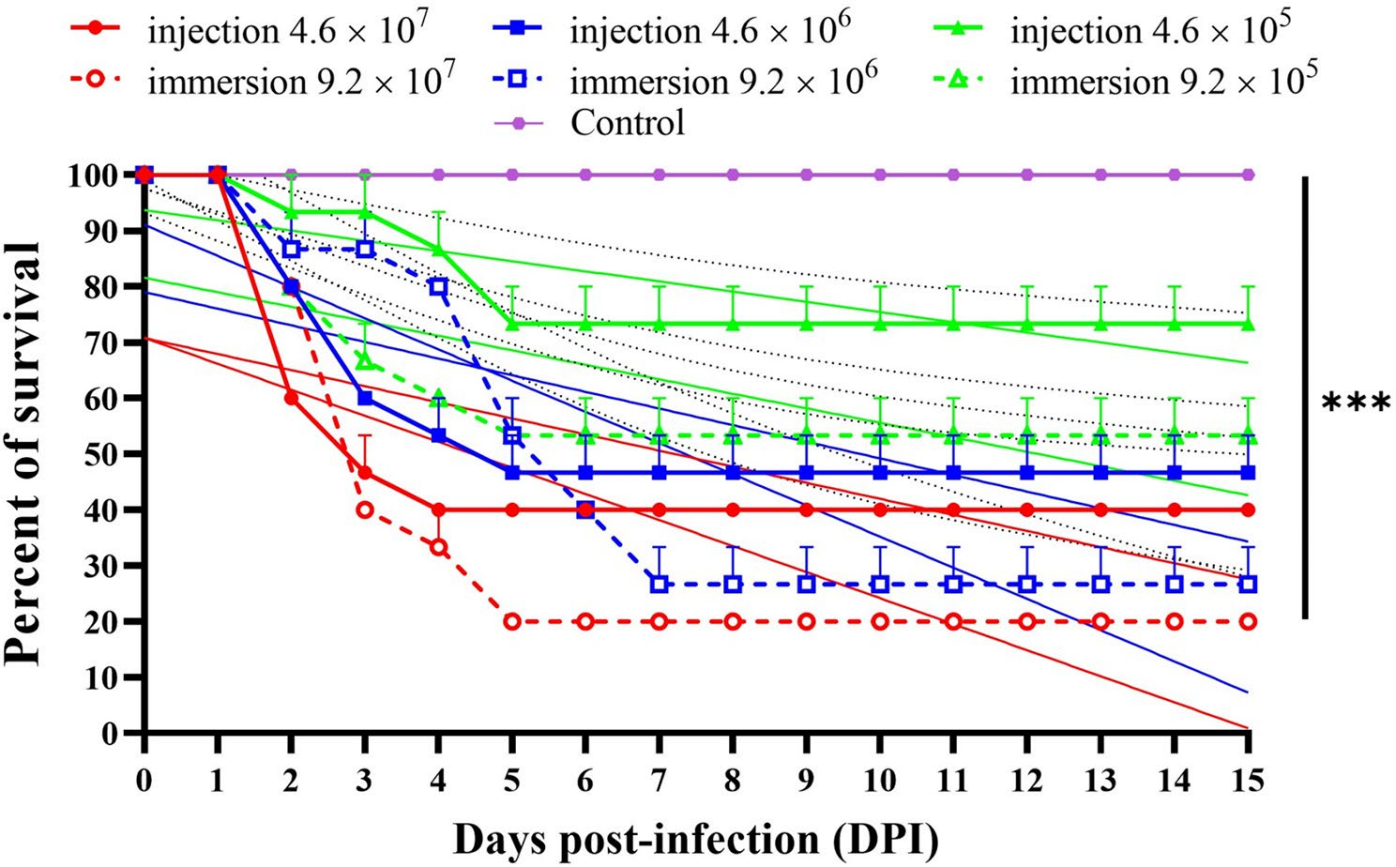
*Streptococcus agalactiae* (MW599202) pathogenicity to Nile tilapia (*Oreochromis niloticus*) after 0.05 ml of phosphate-buffered saline (PBS) containing 4.6 × 10^7^, 4.6 × 10^6^, and 4.6 × 10^5^ CFU was injected intraperitoneally, or after 1 h of immersion in tank water containing 9.2 × 10^7^, 9.2 × 10^6^, 9.2 × 10^5^ CFU/ml. Control groups either received an IP injected of 0.05 ml of sterile PBS (sham control), or they were left untreated as an absolute control. The survival rates were analyzed using GraphPad Prism 8 (version 8.4.3 (686) June 2020)’s simple linear regression

**Fig. 9:**
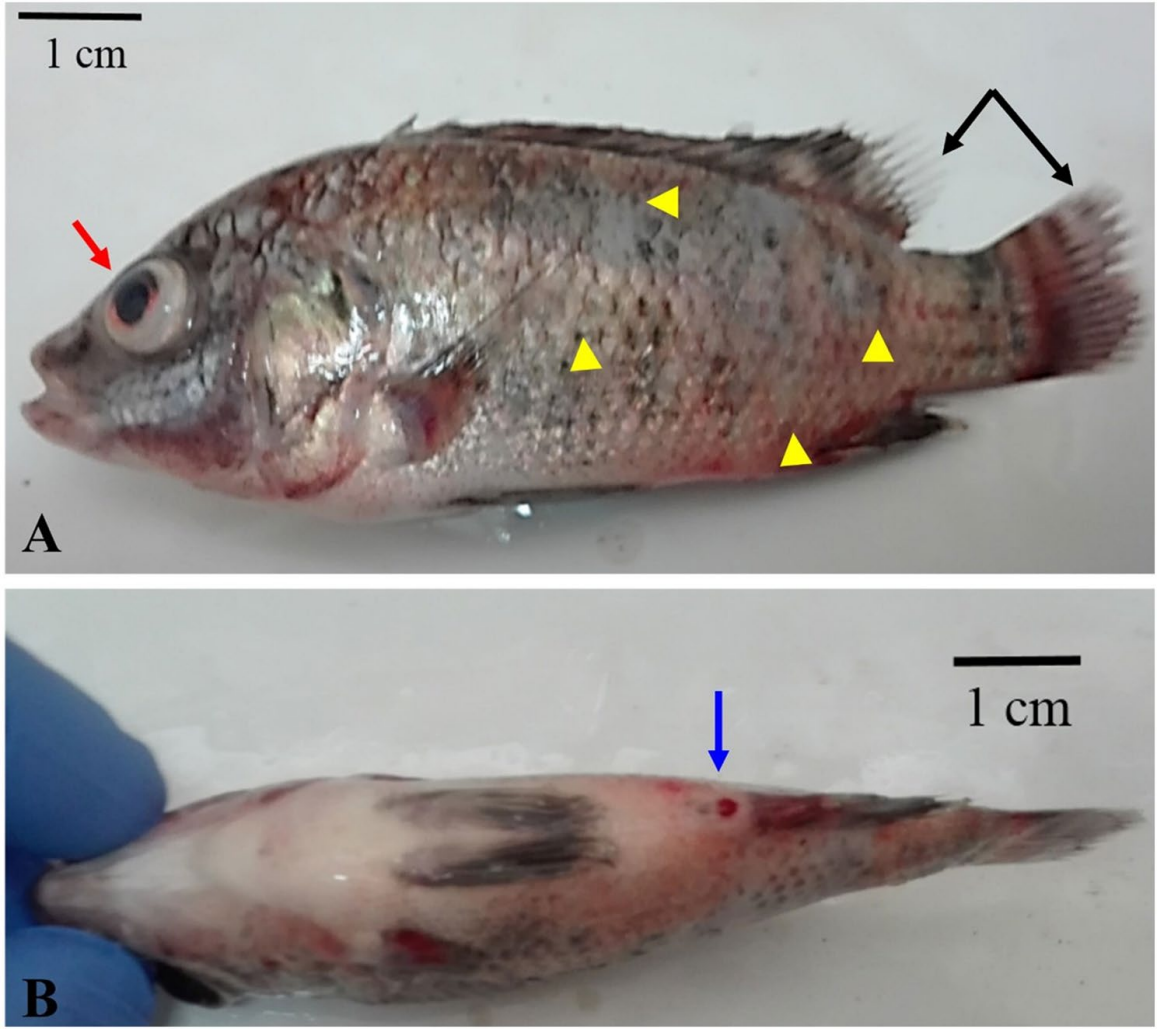
Nile tilapia (*Oreochromis niloticus*) intraperitoneally injected with 0.05 ml of phosphate-buffered saline (PBS) containing 4.6 × 10^6^ CFU of *Streptococcus agalactiae* (MW599202), showing **A**: ocular bleeding (red arrow); severe skin ulcer (yellow arrow-head); frayed dorsal and caudal fin (black arrow) with general petichae throughout the body, and **B**: hemorrhagic vent (blue arrow)

The findings of the pathogenicity test revealed that *S. agalactiae* in *O. niloticus* had remarkable virulence, and the administration method and dosage had an impact on the survival rate (Fig. 8). The mortalities started in all of the infected groups on the second day post-infection (dpi). When *S. agalactiae* was IP injected at a high dose (4.6 × 10^7^ CFU/fish), rapid onset of significant mortalities occurred, resulting in a 26.67% survival rate within 4 dpi. However, a protracted survival rate with a greater survival percentage was seen with IP injection of a low dose (4.6 × 10^5^ CFU/fish), resulting in a survival rate of 53.3% after 5 dpi. The waterborne infection approach, on the other hand, was more virulent and had poorer survival rates across all doses applied. A considerably low survival rate of 20% was attained at 5 dpi with a high immersion dose of 9.2 × 10^7^ CFU/ml. The curve was prolonged to the seventh dpi when 9.2 × 10^6^ CFU/ml was utilized, and a significant survival rate (26.67%) was attained. With a low immersion dose (9.2 × 10^5^ CFU/ml), the survival rate was 46.67%. There were no recorded mortalities in the control groups. Clinical signs included upside-down swimming, lethargy, appetite loss, dark skin pigmentation with hemorrhages and ulceration, frayed fins, and intra- and peri-orbital hemorrhages in all groups of experimentally infected *O. niloticus*. Internally, serosanguinous abdominal fluid, splenomegaly, and liver and brain hemorrhages were observed (Fig. 9).

### Biofilm detection and quantification

**Fig. 10:**
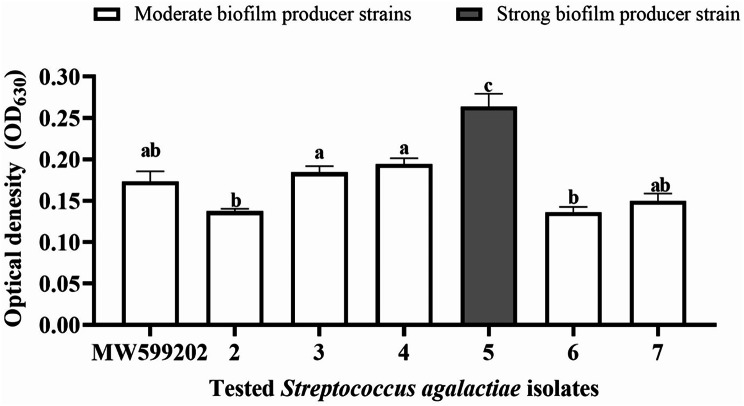
Biofilm formation assay of *Streptococcus agalactiae* (*n* = 7) isolated from Nile tilapia *Oreochromis niloticus* mass mortalities using the crystal violet method. Using one-way ANOVA (Kruskal-Wallis test), different letters showed a significant difference. The gray column denotes a robust biofilm-producing strain of *S. agalactiae*, while the white columns show bacterial isolates that are moderate biofilm-producers

The crystal violet technique, used in the current experiment for biofilm identification and quantification, revealed that all of the tested isolates (*n* = 7) developed biofilm after 48 h of incubation at 28 °C. The majority of the isolates (6 out of 7) were moderate biofilm producers with OD values up to four times higher than the negative control. Only one isolate was identified as a strong biofilm producer with an OD value greater than four times that of the negative control (Fig. 10).

### Antimicrobial activity of medicinal plants

Among the nine different medicinal plants’ ethanolic leaf extracts, all of them were thought to have varying degrees of antibacterial activity against the isolated *S. agalactiae* (MW599202) (Table 3). The most potent antibacterial activity was seen in ethanol leaf extracts of *L. camara*, which also had the lowest MBC (0.24 mg/ml) and MIC (0.12 mg/ml) values. Following closely was *A. caffra*, with MBC and MIC values of 0.485 mg/ml and 0.24 mg/ml, respectively. With MBC and MIC values of 1.95 and 0.976 mg/ml, respectively, the antibacterial activity of the ethanolic leaf extracts from *S. terebinthifolius* and *M*. *communis* was equal. *Ruta graveolens* followed with MBC and MIC values of 3.9 and 1.95 mg/ml, respectively. The ethanolic leaf extracts of *D. viscosa* and *F. nitida* had moderate antibacterial activity, with MBC values of 15.625 and 62.5 mg/ml and MIC values of 7.8 and 31.25 mg/ml, respectively. However, the ethanolic leaf extracts of both *A. indica* and *O. europaea* only mildly inhibited the tested strain, with MBC and MIC values of 125 and 62.5 mg /ml, respectively.

**Table 3:** Minimum bactericidal concentration (MBC), and minimum inhibitory concentration (MIC) of the tested ethanolic leaf extracts of nine medicinal plant against *Streptococcus agalactiae* (MW599202) isolated from cultured Nile tilapia (*Oreochromis niloticus*) mass mortalities

Plant extract	MBC	MIC (% reduction)
	mg ml^− 1^	
*Lantana camara*	0.24	0.12 (99.99%)
*Aberia caffra*	0.49	0.24 (99.99%)
*Schinus terebinthifolius*	1.95	0.98 (99.99%)
*Myrtus communis*	1.95	0.98 (99.99%)
*Ruta graveolens*	3.9	1.95 (99.99%)
*Dodonaea viscosa*	15.63	7.8 (99.99%)
*Ficus nitida*	62.5	31.25 (99.99%)
*Azadirachta indica*	125	62.5 (99.99%)
*Olea europaea*	125	62.5 (99.99%)

### Antibacterial effect of zinc oxide nanoparticles

**Fig. 11:**
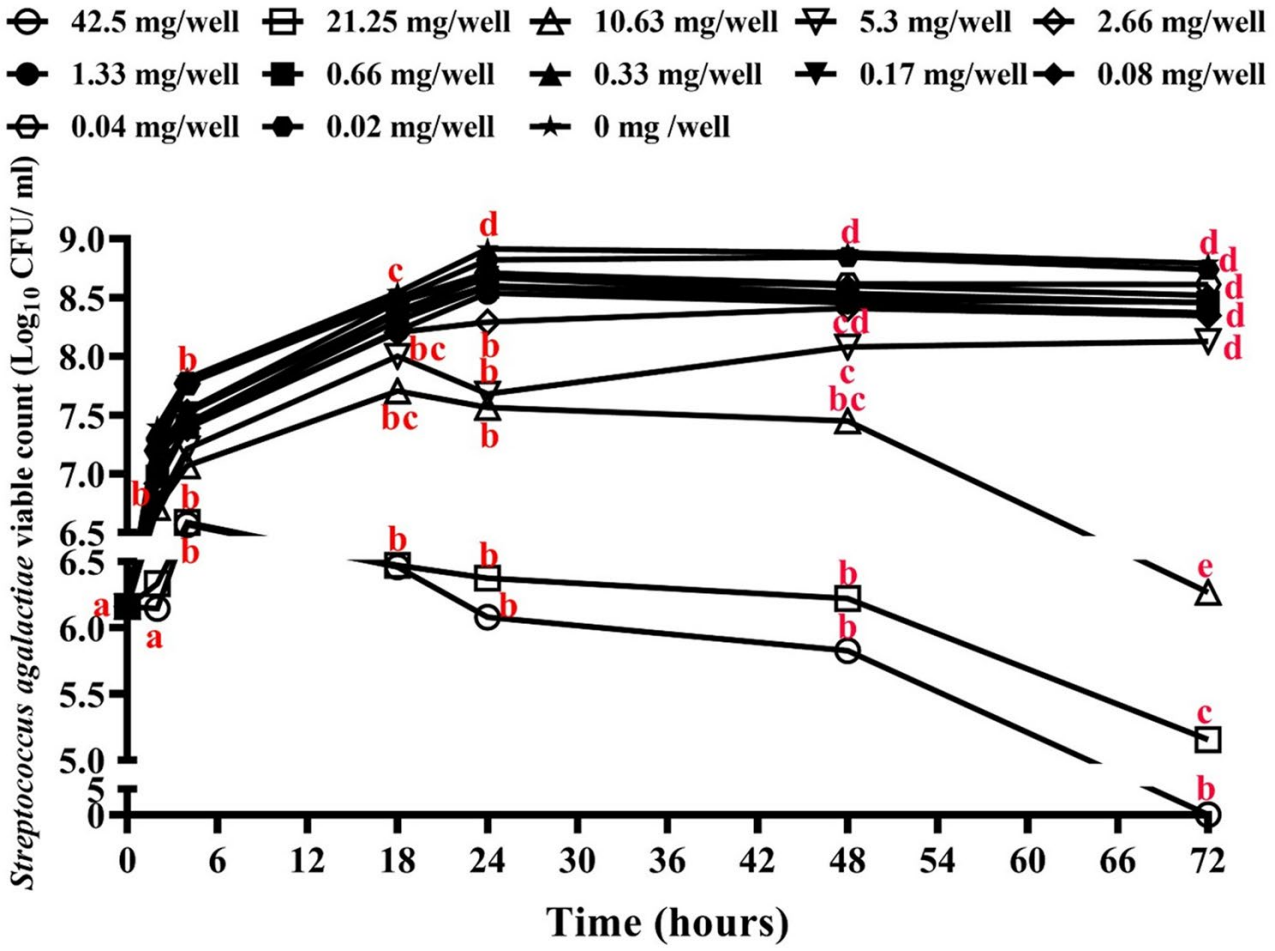
Antibacterial properties of zinc oxide nanoparticles (ZnO NPs) against a tested strain of *Streptococcus agalactiae* (MW599202). Using two-way ANOVA, different letters at the same time point indicate significant differences between tested groups (*P* < 0.001)

In this study, the antibacterial activity of ZnO NPs was assessed using the viable cell counts of *S. agalactiae*. After a 72-hour incubation period, 42.5 mg/well of ZnO NPs eliminated the viable bacterial count representing the MBC, where the bacterial count reduced from 6.2 log_10_ (the starting point) to reach zero CFU/ml at the end of the incubation period, compared to 8.8 log_10_ in the control group. However, after 72 h of incubation with 21.25 mg/well of ZnO NPs, the MIC resulted in a 99.9% reduction in the initial viable bacterial count, dropping from 6.2 to 5.1 log_10_ CFU/ml in the treated group (Fig. 11).

## Discussion

*Streptococcus agalactiae* has emerged as the predominant species of streptococci linked to fish disease, notably in cultured tilapia and even in broodstock as asymptomatic subclinical carriers [41]. It has caused considerable mortalities throughout the world, including in Brazil [11], China [15], Indonesia [14], Thailand [42, 43], Taiwan [44], Malaysia [1], Saudi Arabia [8], and Egypt [9]. Although the disease has been recorded in Egypt, its presence in the southern region remains unclear.

In the current study, *S. agalactiae* huge mortalities were reported in late summer in Assiut city, which is situated in southern Egypt at a latitude of 27°11′00″ North and 31°10′00″ East, known for its extremely hot summers. Global warming-related high water temperatures, intense husbandry, and high stocking densities appear to be risk factors for *S. agalactiae* outbreaks [11]. *Streptococcus agalactiae*’s virulence factors, such as an increase in the thickness of the capsular polysaccharide and its hemolytic activity, were significantly induced, and the number of bacterial loads in fish organs dramatically increased when *O. niloticus* was challenged by IP injection of *S. agalactiae* and kept at a high water temperature of 35ºC, resulting in a high mortality rate [45]. Similar findings were reported by MNA Amal, MZ Saad, AS Zahrah and AR Zulkafli [1] who discovered that poor water quality can affect a fish’s susceptibility to *S. agalactiae* infection in cage-cultured red hybrid tilapia, *O. niloticus*× *O. mossambicus*. Additionally, AH Al-Harbi [8] found that during the summer, when water temperatures rose above 28 °C in combination with high fish stocking densities and poor water quality, there were mortality rates of 40–80% in cultured hybrid tilapia *O. niloticus*× *O. aureus*.

In the current investigation, we were able to isolate *S. agalactiae* from anterior kidney tissue and brain samples. Due to the anterior kidney`s abundance of blood and capillaries, the bacterial load of *S. agalactiae* using qPCR analysis was substantially higher in this organ than in the other organ, indicating that *S. agalactiae* is mostly found in tilapia blood [16]. Furthermore, *S. agalactiae* can continue to survive and proliferate after being phagocytosed by macrophages, indicating that macrophages may act as “Trojan horses” or pathogen carriers to evade the immune system, break the blood-brain barrier, and enter the brain causing meningitis [16]. Moreover, the selection of the brain was based on *S. agalactiae`s* neurotropism, which allows the pathogen to host this relatively unprivileged immune site [41]. Additionally, several experimental studies on *S. agalactiae*’s pathogenicity in *O. niloticus* have revealed that the main target organs should be the brain and kidney tissues [12].

The current study isolates were identified using phenotypic and biochemical testing, and their identity as *S. agalactiae* was further validated using DNA sequencing, *16S rRNA* gene BLAST analysis, as well as *16S rRNA* region amplification using *S. agalactiae-*specific primers. These phenotypic traits substantially resembled those that had previously been isolated from cultured *O. niloticus* [8, 15]. However, *S. agalactiae* isolates (*n* = 13) from 29 *O. niloticus* farms in Brazil were found to have considerable variation in their biochemical profiles [11]. Interestingly, in the current study, isolates were unable to grow at 6.5% NaCl. The same findings were reported by AH Al-Harbi [8] and K Wang, D Chen, L Huang, H Lian, J Wang, D Xiao, Y Geng, Z-x Yang and W-m Lai [15]. In contrast, the Indonesian strain of *S. agalactiae* that was isolated from cultured *O. niloticus* was able to thrive at a NaCl concentration of 6.5% [14]. Surprisingly, the current investigation’s *S. agalactiae* isolate (MW59202) was successfully re-isolated from sterile freshwater inoculated with the pathogen for up to 80, 160, and 160 days post-inoculation and incubation in static conditions at 35, 28, and 15ºC, respectively. This occurred without the presence of its host or any nutritive material. This could be because of robust biofilms` ability to create efficient mechanisms for their environmental persistence, as demonstrated by the current study and the research by TI Heckman and E Soto [46] and AI Isiaku, MY Sabri, MY Ina-Salwany, MD Hassan, PN Tanko and MB Bello [47]. When identical tubes were incubated at the same temperature (28ºC) but with light agitation, no noticeable changes were found (data not shown). Additionally, at an incubation temperature of 15 °C, the greatest colony size was observed. The fact that the size of the recovered colonies at 35 °C was noticeably less than that of colonies incubated at 28 °C indicates that this temperature (35 °C) is a hostile environment for the inoculated pathogens. In the same way, *S. agalactiae* could be successfully isolated from all water samples tested at the Broadstock *O. niloticus* indoor culture facility with water temperatures below 27 °C that favored pathogen replication [41].

Among several methods that aid in bacterial identification and profiling, including single-strand conformation polymorphism (SSCP), high-resolution melt analysis (HRMA), repetitive element palindromic PCR (REP), restriction fragment length polymorphism (RFLP), terminal restriction fragment length polymorphism (T-RFLP), amplified fragment length polymorphism (AFLP), denaturing gradient gel electrophoresis (DGGE), pulsed-field gel electrophoresis (PFGE), DNA microarray, propidium mono-azide PCR (PMA), RAPD [48], and the most recent Whole Genome Sequencing (WGS) Analysis, are still in use. However, RAPD analysis is still utilized as a simple and reliable method for showing the genetic diversity among the tested samples [49]. RAPD molecular analysis is considered one of the top techniques [48]. It is a quick and accurate technique that possesses adequate discriminatory power for establishing the genetic similarity of *S. agalactiae* isolates derived from various fish samples to trace the potential infection source. The RAPD approach is more popular in profiling research than restriction fragment length polymorphism (RFLP) due to its ease of use, affordability, lack of technical labor requirements, speed, and the required amount of DNA. However, there are negligible differences between the two when considering the level of polymorphism, the accuracy of genetic distance estimates, and the test’s statistical power [50]. The disadvantage is the low fingerprint reproducibility, which necessitates stringent PCR condition standardization. Variations in primer ratios, annealing temperatures, and DNA polymerase concentrations can all result in variations in the final results [51]. The limitation of using RAPD is the poor reproducibility of fingerprints, as it requires strict standardization of reaction parameters [48]. In the current investigation, *S. agalactiae* isolated from moribund *O. niloticus* were collected and studied using RAPD analysis. Despite using six distinct primers, each with a different G + C content, all of the *S. agalactiae* isolates (*n* = 4) examined displayed the same RAPD patterns, indicating their shared origin. This could be because the host and the location being the same. The *S. agalactiae* strains acquired from golden pompano (*Trachinotus blochii*) differed genetically, based on RAPD genetic analysis, from those isolated from *Oreochromis sp.* in Malaysia, according to MN Amal, M Zamri-Saad, A Siti-Zahrah, AR Zulkafli and M Nur-Nazifah [52] classification of 181 *S. agalactiae* strains into 13 groups based on the origin of their geographical location. Furthermore, *S. agalactiae* isolates from sewage water and moribund Mullet (*Liza kluningeri*) mass mortalities in Kuwait Bay were clustered together, suggesting a common origin [53].

Challenge tests are an essential tool for diagnosing diseases in fish caused by microbial pathogens. These tests involve exposing fish to a known quantity of the suspected pathogen in a controlled environment to observe their response, determine the presence of the disease and assess the virulence of the pathogen under controlled conditions. This information can help understand the dynamics of disease outbreaks and design effective disease management strategies. In the pathogenicity experiment of the present study, isolate (MW599202) caused a high cumulative mortality rate of up to 80% with aberrant swimming behavior, a classical indicator of brain infection, and meningoencephalitis, a trait unique to *S. agalactiae* infection. In waterborne infection trials that mimic the natural infection strategy, the current study isolate was sufficiently virulent to generate high mortality rates of 80 and 73.3% within 5 and 7 dpi at high and moderate dosages, respectively. The primary routes of disease transmission in the culture system appeared to be indirect contact through contaminated water and direct contact between healthy fish and sick or dead fish in the natural environment [1, 11, 54]. Given that *S. agalactiae* has recently been found to be able to infect the CNS via the nose-to-brain pathway [55], this may be caused by *S. agalactiae*’s neurotropism. Furthermore, a high mortality rate (73.3%) was observed following IP injection of the tested isolate at a high dose (4.6 × 10^7^/fish), which was not different from the high immersed dose (4.6 × 10^7^ CFU/ml). This could be because of the capsular polysaccharide, allowing *S. agalactiae* to evade early phagocytosis and bacterial killing by the host immune response [45] as well as to multiply in fish blood, which in turn causes their survival, systemic proliferation [54], and disease occurrence with high mortality rates that were noticed in the current research. The pathogen could be isolated from the surviving survivors, indicating that they may have developed into subclinical carriers that could develop an overt infection-causing disease in response to unidentified stressors [18] or vertically transmit the pathogen to their offspring [41].

A biofilm is a community of bacteria that is embedded in an organic matrix and adheres to a surface. Adopting such a unique lifestyle has advantages such as protection from desiccation, nutritional concentration, escape from antibacterial medications, and host immune responses [22]. This might be due to the bacterial capsule, which is a key component of biofilm formation [56]. In the current investigation, it was discovered that all of the tested isolates (*n* = 7) generated biofilms to varying degrees. One isolate was a powerful biofilm generator, while six of the isolates produced moderate amounts of biofilm. This suggests that these isolates can colonize any submerged surfaces in the aquatic environment and can be a continual source of infection for other aquatic animals that are vulnerable to infection [57]. Additionally, *S. agalactiae* has been shown to form biofilms in the brain tissues and surrounding meningeal surfaces of tilapia, suggesting that biofilms may also be involved in the pathogenesis of this pathogen. These biofilms make bacteria more resilient to the immune systems of the host while facilitating their passage through the blood-brain barrier, possibly via a “Trojan horse” mechanism [47]. Additionally, the brain can remain infected long after the bacteria have been eliminated from other organs due to the humoral response’s importance in eradicating the bacteria and antibodies’ difficulty in crossing the blood-brain barrier [58]. This makes the fish both a carrier and a reservoir of infection.

The in vitro antibacterial activity of the tested plant extracts differed significantly against the tested strain of *S. agalactiae*, with *L. camara* and *A. caffra* being the most potent and promising results *S. agalactiae* is a Gram-positive bacterium with a cell wall structure consisting of a single-layer structure [59], suggesting that the antimicrobial activity of *L. camara* leaves may be attributed to the presence of cardiac glycosides, flavonoids, saponins, tannins, and terpenoids [60]. Furthermore, the essential oil content, phenols, flavonoids, xanthones, and lectin contained in *S. terebinthifolius* leaf extract may be responsible for its potent antibacterial activities [61, 62]. According to A Ghandour, M Abdel-Rahim, SAL Bayoumi, HM Sayed and O El-Badawy [39], *D. viscosa* leaf extract had antibacterial activity against the tested Gram-positive cocci.likely due to the presence of 5,6,8-trihydroxy-7,4-dimethoxyflavone in the extracted leaves [63]. In addition, phytochemical studies have attributed the following phytochemicals to the reported antibacterial limonoids such as mahmoodin and tetranortriterpenoids like azadirone, epoxyazadiradione, nimbin, gedunin, azadiradione, deacetylnimbin, 17-hydroxyazadiradione, and the protolimonoid, naheedin [64]. Fortunately, promising in vivo results have been reported using herbal plant extracts [65–67]. S Bhuvaneswari, J Sivakumar, CJ Lora, S Suriyakodi and S Venu [65] found that intramuscular injection of Mrigal (*Cirrhinus cirrhosus*) fingerlings with ethanolic leaf extract of *L. camara* followed by *Aeromonas hydrophila* challenge, yielded a promising antibacterial effect compared to untreated fish. Furthermore, immersion of *S. terebinthifolius* essential oil in water for 8 days was found to support memory formation through the inhibition of acetylcholinesterase activity and decreasing oxidative stress in scopolamine-treated zebrafish brains [67]. Additionally, feeding rabbitfish (*Siganus fuscescens*) with stem and leaf extract of *Scutellaria baicalensis* demonstrated a strong antibacterial effect. This inhibited the growth of pathogenic bacteria like *Deltaproteobacteria* and *Fusobacteria*, while also promoting the growth of probiotics such as *Erysipelotrichia* [66].

Nanoparticles are extensively utilized in various fields in our daily lives, including cosmetics, environmental catalysts, electronics, and biomedical and medical applications. This is due to their antibacterial, antifungal, anti-inflammatory, and wound-healing properties [68]. Furthermore, studies have shown that dietary nanoparticles can enhance growth indices, survival, and feed utilization in different fish species [69]. For instance, supplementation with ZnO-NPs has been found to improve the growth performance of zebrafish by regulating oxidative stress and the expression of growth-related genes [70]. In the present work, ZnO NPs demonstrated effective antibacterial activity against the examined *S. agalactiae* isolate (MW599202). This bactericidal action was found to be enhanced with increasing nanoparticle concentrations and/or exposure periods, as previously reported by I Ahmad, MY Alshahrani, S Wahab, AI Al-Harbi, N Nisar, Y Alraey, A Alqahtani, MA Mir, S Irfan and M Saeed [71]. For instance, exposure to 42.5 mg/well induced an inhibition of the viable cells that reached zero CFU/ml 72 h post-exposure. Additionally, 72 h after the exposure to 21.5 mg/well of ZnO NPs, caused a 99.9% reduction in the viable bacteria. This could be the consequence of the bacterial cells and ZnO NPs coming into close contact, which caused the cell wall to become disorganized and more permeable, ultimately rupturing the cell wall [72]. Furthermore, after 24, 48, and 72 h of incubation at 28ºC, the viable cell counts were considerably lower than the control with the highest three concentrations (42.5, 21.25, 10.63 mg/well). This might be the result of the liberation of H_2_O_2_, which is one of the likely mechanisms for ZnO NPs’ bactericidal action [73], as well as an increase in intracellular reactive oxygen species (ROS) production [74] which interacted with polyunsaturated fatty acids, a component of lipid bilayer, and produced malondialdehyde (MDA) and lipid peroxidation disrupting the integrity of the bacterial cell membrane [75, 76]. This results from the lipid peroxides forming reactive molecules that start oxidative stress and harm the bacterial cells [76]. In addition to lipid peroxidation, the production of ROS influences protein alteration, enzyme inhibition, bacterial cell macromolecule destruction, and damage to nucleic acid [77]. However, further investigation is still needed to examine the in vivo antibacterial effect of ZnO NPs against *S. agalactiae* infection in *O. niloticus*.

## Conclusion

In conclusion, the current research was able to isolate *S. agalactiae* from mass mortalities of *O. niloticus* in a fish farm in Assiut, Egypt. Phylogenetic and RAPD analysis revealed that the isolates were similar and shared genetic traits with other *S. agalactiae* strains that had been isolated from various sources, including human samples, raw milk (type strain), different fish hosts and locations all over the world. Once fish are infected, they can disseminate the pathogen in the surrounding environment which could be isolated in the current research for approximately three months at an incubating temperature of 35 ºC. This indicates the pathogen´s ability to persist in fish environments and its capability to escape from unfavorable environmental conditions by forming biofilms, making it difficult to eliminate from an aquaculture facility using an intergenerational approach. In vitro studies were conducted to explore pathogen control using zinc oxide nanoparticles and ethanolic leaf extracts from medicinal plants, which showed promising results. However, further research is necessary to assess their safety and effectiveness in vivo.

## Data Availability

Data and materials are available upon reasonable request from the corresponding author. The datasets generated during the current study are available in GenBank under accession number MW599202.
